# Identification of new resistance loci against wheat sharp eyespot through genome-wide association study

**DOI:** 10.3389/fpls.2022.1056935

**Published:** 2022-12-12

**Authors:** Xujiang Wu, Junchan Wang, Di Wu, Wei Jiang, Zhifu Gao, Dongsheng Li, Rongling Wu, Derong Gao, Yong Zhang

**Affiliations:** ^1^ Institute of Agricultural Science of the Lixiahe District in Jiangsu Province, Yangzhou, China; ^2^ Key Laboratory of Wheat Biology and Genetic Improvement on Low and Middle Yangtze River Valley Wheat Region, Ministry of Agriculture, Yangzhou, China; ^3^ Jiangsu Co-Innovation Center for Modern Production Technology of Grain Crops, Yangzhou University, Yangzhou, China; ^4^ Jiangsu Key Laboratory of Crop Genomics and Molecular Breeding, Yangzhou University, Yangzhou, China

**Keywords:** wheat, sharp eyespot, genome-wide association study (GWAS), quantitative trait loci (QTL), single nucleotide polymorphism (SNP)

## Abstract

**Introduction:**

Wheat sharp eyespot caused by Rhizoctonia cerealis is a serious pathogenic disease affecting plants. The effective strategy for controlling this disease is breeding resistant cultivar. However, to date, no wheat varieties are fully resistant to sharp eyespot, and only a few quantitative trait loci (QTLs) have been shown to be associated with sharp eyespot resistance.

**Methods:**

To understand the genetic basis of this disease, a genome-wide association study (GWAS) of sharp eyespot resistance in 262 varieties from all China wheat regions was conducted.

**Results:**

After cultivation for three years, only 6.5% of the varieties were resistant to sharp eyespot. Notably, the varieties from the middle and lower Yangtze River displayed higher sharp eyespot resistance than those from Huanghuai wheat zone. Only two varieties had the same resistance level to the control Shanhongmai. The results of GWAS showed that 5 single nucleotide polymorphism (SNP) loci were markedly related to sharp eyespot resistance in the three years repeatedly, and two QTLs, qSE-6A and qSE-7B, on chromosome 6A and 7B were identified. Based on the ‘CG’ haplotypes of significant SNPs, we found that the two QTLs exhibited additive effects on attenuating sharp eyespot resistance.

**Discussion:**

These results provide novel insights into the genetic basis of sharp eyespot resistance in China wheat varieties. The SNPs related to sharp eyespot resistance can be applied for marker-assisted selection in plant breeding.

## Introduction

Bread wheat (*Triticum aestivum* L.) is an essential cereal crop cultivated worldwide, and is the main source of calories for the growth of global population (Gupta et al., 2020). Sharp eyespot leads to wheat yield losses, thereby posing serious threats to global food security. The extensive uses of nematicides and fumigants are not recommended because of environmental concerns. Breeding resistant varieties is considered the most effective and economical strategy for controlling plant diseases compared with pesticides and nematicides. To date, no wheat germplasm is fully resistant to sharp eyespot, and only a few varieties demonstrate partial resistance, including CI12633, Luck and AQ24788-83 (Chen et al., 2013; Wu et al., 2017).

The development of sharp eyespot disease was affected by the environmental conditions (Glynne, 1950). Several environmental factors are related to sharp eyespot, such as temperature, sowing time, soil-related factors, nutrient availability and agronomic practices (Hamada et al., 2011). Most scholars agreed that the high disease incidence in early sown fields can be attributed to the prolonged period of *Rhizoctonia cerealis* infection before winter. The favorable temperature of sharp eyespot is 16-28°C. If the temperature gets too high or too low, it will slow down the incidence of the disease. Some studies found that the disease severity was affected by soil fertilizers (Colbach et al., 1997). At first, many elements (the environments, the experiments repeat or the standard of disease sore) affected the phenotype of the disease, some researchers thought that the sharp eyespot was controlled by one or two major genes (Cai et al., 2006).

An accurate method for detecting quantitative traits loci (QTLs) in sharp eyespot resistance is a vital precondition for wheat sharp eyespot resistance studies. To date, the major methods for assessing sharp eyespot resistance include seeded inoculation, colonized wheat kernels on the soil surface (Cai et al., 2006; Wu et al., 2017), screening a disease nursery (Li et al., 2013), two-step colonized wheat kernels (Liu et al., 2020) and toothpick inoculation (Cai et al., 2006; Liu et al., 2020). In this study, we chose the spreading of colonized wheat kernels on the soil surface inoculation method, and moisturized by fog. Although this method greatly increases the repeatability, and is labor-intensive and time-consuming, it can increase the degree of accuracy for the phenotyping (Wu et al., 2017).

With the development types and quantity of molecule markers, most scientists discovered that sharp eyespot in wheat was a standard quantitative trait, which can be controlled by QTLs or multiple genes (Chen et al., 2013; Wu et al., 2017; Guo et al., 2017; Liu et al., 2021; Su et al., 2021). Ren Lijuan (Lijuan et al., 2004) first identified sharp eyespot resistance QTLs using SSR markers. Following that, approximately 30 QTLs of sharp eyespot resistance have been discovered on the wheat 21 chromosomes, except 2A, using recombinant inbred lines (RILs) mapping populations. In addition, Chen Jiang et al. (Chen et al., 2013) performed an association mapping with 549 simple-sequence repeat DNA markers using 241 RILs, and identified seven QTLs significantly contributed to sharp eyespot resistance. Wu Xujiang et al. (Wu et al., 2017) detected five QTLs significantly associated with sharp eyespot resistance through association analysis with the Illumina iSelect 90K SNP wheat chip and 101 SSR markers using 224 RILs. Liu Caiyun et al. (Liu et al., 2021) identified two stable QTL and three novel stable QTL through association analysis with the polymorphic Affymetrix Axiom™ 55K SNPs using 215 F_8_ lines from Niavt14/Xuzhou25. Over a period of twenty years, the discovered resistance genes were about thirty. However, only a few of these QTLs can be applied for sharp eyespot resistance. Moreover, no QTL for sharp eyespot resistance is fine-mapped and has been cloned so far by the map-based cloning method.

At present, all the mapping population of sharp eyespot research is RILs. However, this approach is time consuming, as the QTL mapping may require a large biparental population. Genome-wide association studies (GWAS) have become a crucial method to detect putative genes for complex traits in plants. Compared to the method in sharp eyespot study, GWAS employs high-density single nucleotide polymorphism (SNP) markers and more diverse natural populations, which can identify QTLs much closer to the putative genes and determine favorable haplotypes of sharp eyespot resistance among tested varieties.

In this research, 262 wheat cultivars were genotyped using the 90K SNP array, involving a large number of wheat cultivars from all China wheat regions. According to the multi-environmental trail data, GWAS was employed to identify favorable alleles of sharp eyespot. The results provide new insights into marker-assisted selection in plant breeding.

## Materials and methods

### Wheat materials

An association panel comprising 262 wheat cultivars was used for SNP genotyping and 3-year (2018, 2019 and 2020) sharp eyespot resistance phenotyping (**Supplemental Table 1**). Of them, 43 cultivars were collected from the middle and lower reaches of Yangtze River, 21 from Huanghuai wheat zone, 5 cultivars were derived from foreign regions, and 7 cultivars were obtained from Shanxi provinces in the Northern China Winter Wheat Region and 15 cultivars from Southern China. Six wheat varieties with known sharp eyespot resistance levels, CI12633 (moderately resistant, the disease index was between 44.8-50.27), shanhongmai (moderately resistant), Yangmai158 (highly susceptible), and Yangmai9 (high susceptible) were served as references in the analysis. The experiments were carried out at Wantou Experimental Station, Institute of Agricultural Sciences of the Lixiahe, Yangzhou, Jiangsu Province, China (latitude 32.24°N, altitude 8 m, annual precipitation approximately 1000 mm, from early November to the next May) for three years.

### Sharp eyespot fungal inoculation

The *R. cerealis* isolate R0301, a predominant strain in Jiangsu province, China, was obtained from Prof. Shibin Cai and Prof. Huaigu Chen (Jiangsu Academy of Agricultural Sciences, China). Before use, R0301 was inoculated on potato-dextrose agar (PDA). Inoculum was generated on the sterilized wheat kernels as described previously (Chen et al., 2013).

Randomized complete block designed with 3 replicates/environment was used in this study. The cultivars (50 seeds/row) were sown in one row of 80*40 cm for each replication. Some seedlings were removed to obtain a density of 40 cultivars/row. The inoculum method was the same with Wu Xujiang et al. (Wu et al., 2017). In early March, when the weather became warm and wet, the plants were inoculated by placing R0301-colonized wheat kernels on the soil surface close to the plants, but not touching them. The kernels were then covered with earth and water was sprinkled three times a day for the first month in non-raining days. Overall, the weather was very suitable for sharp eyespot outbreak two of three seasons with consistent rainfall occurring throughout the experimental period until the final disease recording.

### Phenotyping

In spring next year, when the susceptible mocks Yangmai 9 began to succumb to R. *cerealis* infection, all sample plants except the edge plants were dug up and individually assessed for disease (approximately 200-250 wheat stems of each line). The infection types were categorized qualitatively from 0 to 5: 0, no lesion; 1, the lesion appeared on the sheaths rather than stems; 2, the width of the lesion was <50% of the infected stem perimeter; 3, the width of the lesion is >50 and <75% of the infected stem perimeter; 4, the width of the disease lesion is >75% of the infected stem perimeter; 5, white spike or dead plant. Disease index =((0×X_0_+1×X_1_+2×X_2_+3×X_3_+4×X_4_+5×X_5_)/[(X0+X_1_+X_2_+X_3_ +X_4_+X_5_)×5]) ×100, where X_0_-X_5_ indicated plants with infection types 0-5, respectively (Wu et al., 2017).

### DNA extraction and genotyping

A modified CTAB method described in the CIMMYT laboratory protocol was used to extract genomic DNA from fresh leaf samples (Clarke, 2009). The RNA yield was assessed using a NanoDrop 8000 spectrophotometer. The Illumina iSelect 90K SNP technology (Chinese academy of agricultural sciences, Beijing) was used for genotyping. SNPs with minor allele frequency (MAF) <0.10 and/or with >10% of missing data were eliminated. The SNPs were mapped on wheat chromosomes in accordance with Chinese Spring reference genome sequences (International Wheat Genome Sequencing Consortium (IWGSC), 2018).

### Population structure and linkage disequilibrium analyses

PS was assessed by Bayesian cluster analysis using the Structure 2.3.4 software used 26517 SNPs. There were 34039 SNPs distributed on all 21 wheat chromosomes with *r^2^* <0.2 (Najafi et al., 2019). LD was conducted with TASSEL 5.0 software using the expected versus observed allele frequencies of the SNPs (Liu et al., 2017).

### GWAS analysis

The identification of SNPs related to sharp eyespot resistance was performed as described previously (Zhou et al., 2016). The GWAS panel consisted of 90k SNP markers (**Supplemental Table 2**), which was analyzed based on the mixed linear model (MLM) and TASSEL 5.0 software (Bradbury et al., 2007). In the MLM, the kinship matrix is used jointly with PS (Henderson, 1975). To reduce type I error, the region with SNPs (P<1*10^-4^) in a 200-kb genomic window was selected for further analyses. PerL was used to construct Manhattan maps (Christiansen et al., 2012). A standard linear mixed model was fitted by EMMAX (Kang et al., 2010), and then added with 3 principal component covariates. The Manhattan and quantile-quantile plots were generated using the R package (https://cran.rproject.org/web/pachages/qqman/).

### Statistical analysis

Statistical analyses, including ANOVA, heritability, correlation, and descriptive statistics, were conducted using the R statistical package (R Core Team, 2019). The broad-sense heritability for the traits was estimated by the formula *H^2^* =VG/(VG+VE) where VG and VE represent the estimates of genetic and environmental variance, respectively (Arora et al., 2017).

## Results

### Phenotypic variation, correlation, and heritability of sharp eyespot

**Figure 1:**
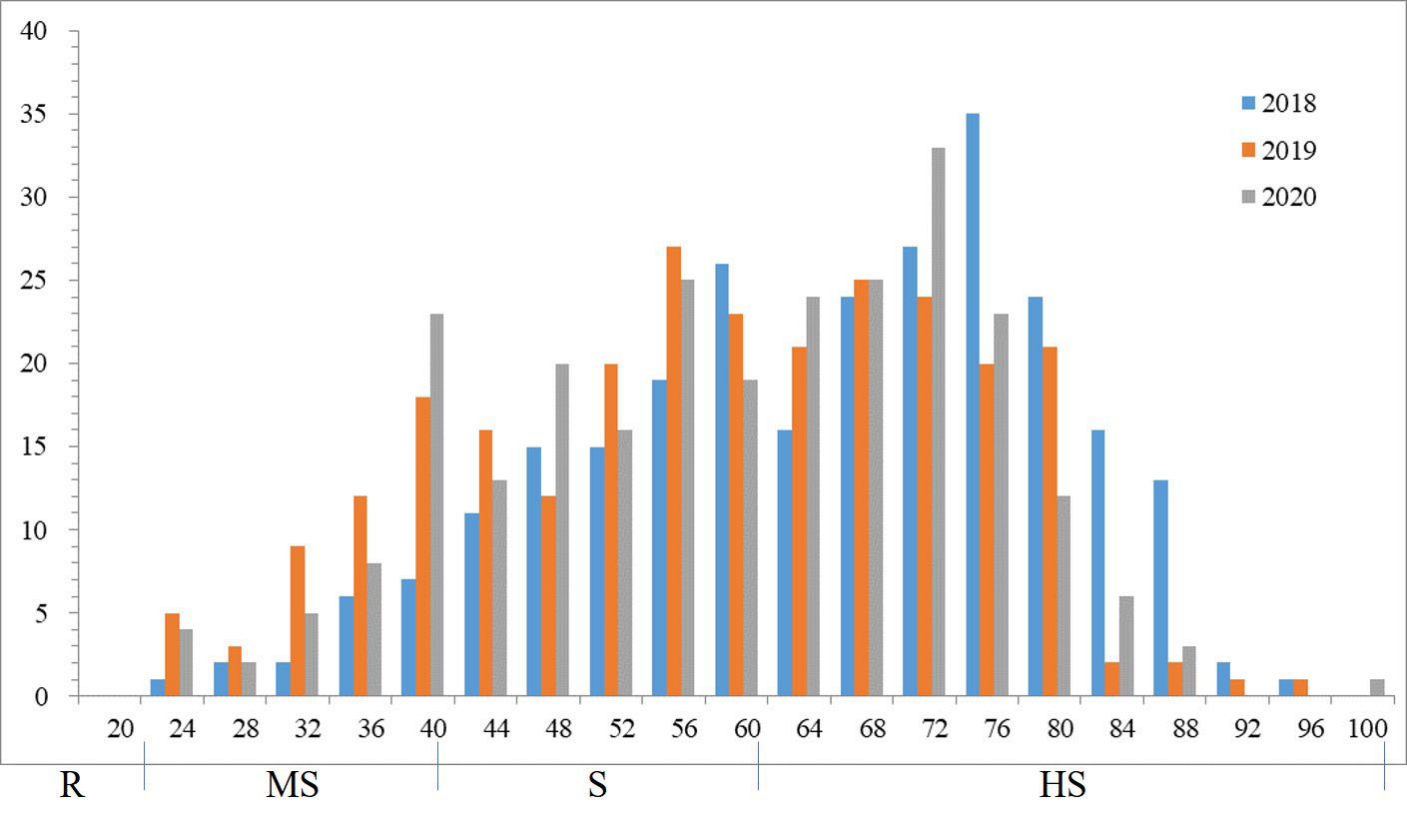
Frequency distributions of the sharp eyespot disease index in 2018, 2019 and 2020.

Phenotypic variations of sharp eyespot among the 262 wheat accessions were evaluated at the experimental field in Yangzhou during the winter seasons of 2018-2020 in MLM. Phenotypic evaluation showed the presence of sharp eyespot resistant in three years. The mean of sharp eyespot disease index ranged from 25.86% to 95.23% (**Supplemental Table 3**), with a normal distribution pattern. The standard deviation and coefficient of variation (CV) were 13.5 and 4.61%, respectively. The frequency of sharp eyespot disease index was normally distributed in each year (**Figure 1**), implying the nature of the disease.

Significant positive correlations were observed among the three years for sharp eyespot disease index (r=0.75-0.95), suggesting that the traits occur consistently (**Table 1**). The severity of the disease index was most high in 2018 growing season (**Supplemental Figure 1**). Wheat cultivars from different wheat regions in China had varying degree of resistance to sharp eyespot. The cultivars from Jiangsu province were consistently resistant to sharp eyespot in three seasons, while those from Henan province had the greatest vulnerability. The ANOVA results revealed significant differences among the genotypes for sharp eyespots resistance (**Table 2**). High broad-sense heritability was observed for the trait, with 0.86, indicating that the trait was largely influenced by genetic factors.

**Table 1:** Correlation coefficients among the three trial environments for the sharp eyespot disease index.

	2018 DI	2019 DI	2020 DI	AVERAGE
2018 DI	1	0.8**	0.75**	0.92**
2019 DI	0.8**	1	0.84**	0.95**
2020 DI	0.75**	0.84**	1	0.92**
AVERAGE	0.92**	0.95**	0.92**	1

**Significant at P < 0.01.

**Table 2:** The ANOVA of wheat sharp eyespot disease scores of the cultivars.

	SS	df	MS	F	P	*H^2^*
Replication	6642.414	2	3321.207	77.31	0	0.86
Cultivar	135458.113	261	518.997	12.081	0	
Error	22424.81	522	42.959			
Total	164525.337	785				

DF, (Degree of freedom); SS, (Sum of Square); MS, (Mean square); H^2^, (Heritability).

The cultivars from different wheat regions had varying degree of resistance to sharp eyespot (**Table 3**). Those from Huanghuai region and foreign were consistently resistant to sharp eyespot in the three seasons, while from the wheat region in the middle and lower reaches of Yangtze River exhibited the greatest vulnerability.

**Table 3:** Sharp eyespot disease scores of different wheat regions.

Wheat region	Average disease score
XIBEI	61.13 ± 2.7
CHANGJIANG	60.90 ± 1.9
ABROAD	60.05 ± 4.3
HUANGHAI	59.80 ± 2.1
XINAN	58.39 ± 2.6

CHANGJIANG, the wheat region in the middle and lower reaches of Yangtze River; XINAN, southwest wheat region; HUANGHUAI: Huanghuai wheat zone; XIBEI: the northwest wheat region; ABROAD: the cultivars come from abroad countries.

Values are Mean ± SD (n = 3).

### Population structure and LD analyses

PC analysis is compulsory in many populations as the presence of a large number of genotypes in the research can result in a higher quantity of false associations between the phenotypes and unlinked markers. A total of 34039 SNPs (**Supplemental Table 4**), of which 19975 were mapped to a relative position on a single chromosome. Among them, 6751, 8248 and 4587 were found in A, B and D genome chromosome, respectively, but 389 SNPs could not be presented in genome chromosome. From the kinship analysis, a break in the slope was observed at *K = 2*, followed by a flattening of the curve (**Supplemental Figure 2A**); hence, it was revealed that the most likely number of sub-populations was two (*K = 2*; **Supplemental Figure 2B**). In addition, the results were verified by principle component analysis (PCA), on account of the standardized covariance of genetic distances of SNP markers (**Supplemental Figure 2C**).

For LD analysis, only SNPs with a known position and a MAF >5% were selected. The LD half-width (the point at which LD decayed to 48% of the peak) was 10.5 cM. The *r^2^* values of A, B and D sub-genomes gradually declined with increasing genetic distance (**Supplemental Figure 3**).

### Identification of new QTLs for sharp eyespot resistance

**Figure 2:**
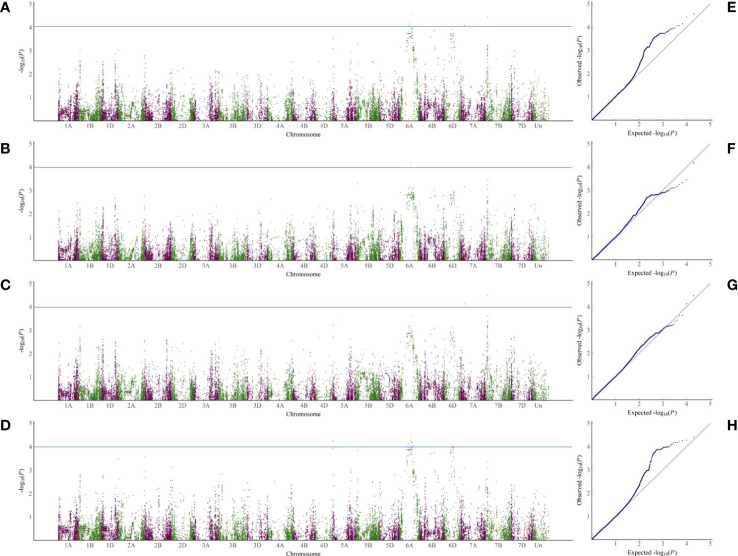
GWAS analysis of SNP loci related to sharp eyespot resistance in the three years. **(A–D)**, Manhattan plots of SNPs related to sharp eyespot resistance on 21 wheat chromosomes in three years and mean of three years. The *x-axis* and *y-axis* represent the genomic coordinates and LOD score for each SNP, respectively. **(E–H)**, Quantile-quantile (Q-Q) plots for the GWAS data in 2018 **(E)**, 2019 **(F)**, 2020 **(G)** and mean of three years **(H)**.

According to the 34039 SNP dataset and the disease index of the selected varieties, 5, 19 and 14 SNPs were markedly related to sharp eyespot resistance in 2018, 2019 and 2020, respectively. The highest number of SNPs in 2019 may be attributed to a large phenotypic variation. The contribution of each SNP on phenotypic variance ranged from 2.7% to 8.5%. These SNPs were distributed mainly on chromosomes 6A, 7A and 7B. Among them, two SNP markers were repeatedly observed in all the three experiments, and three SNPs were detected in two years (**Supplemental Table 5**). These five SNPs were primarily located on chromosome 6A. Due to the high degree of LD in the wheat genome, the SNP clusters identified on chromosome 6A (333.95-351.08 Mb) might represent the chromosome regions with sharp eyespot loci. Although many QTLs were detected, only two regions were found to be large effective QTL for sharp eyespot resistance, which were designated as *qSE-6A* and *qSE-7B*, respectively (**Figure 2**).

### The additive effect of SNP haplotypes related to QTLs *qSE-6A* and *qSE-7B* related SNP haplotypes on sharp eyespot resistance

**Figure 3:**
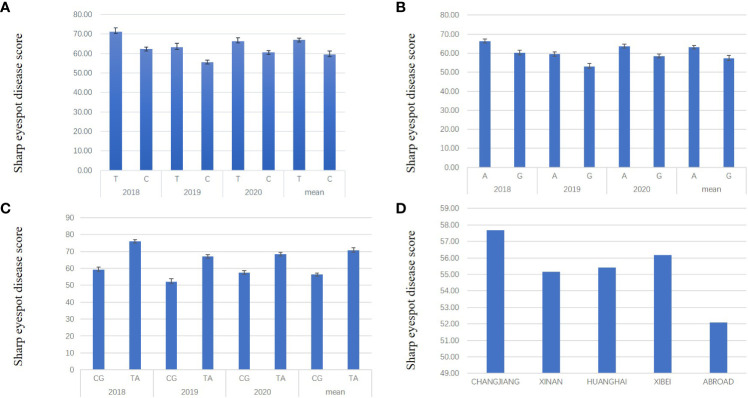
Resistance effects and ‘CG’ haplotype distribution of *qSE-6A* and *qSE-7B.* **(A)**, Effects of the favorable allele ‘C’ of SNP BS00078658_51 (represent *qSE-6A*) versus the unfavorable allele ‘T’. **(B)**, Effects of the favorable allele ‘G’ of SNP wsnp_Ex_c3501_6407527 (represent *qSE-7B*) versus the unfavorable allele ‘A’. **(C)**, Effects of the favorable haplotype ‘CG’ of SNP BS00078658_51 and wsnp_Ex_c3501_6407527 compared to the unfavorable haplotype ‘TA’. **(D)** the ratio of ‘CG’ haplotype varieties in each wheat region.

To assess the effect of QTL on sharp eyespot resistance, the most significantly associated SNPs were used to distinguish varieties with/without the resistant alleles. For *qSE-6A*, BS00078658_51 was the most significant SNP locus, in which ‘C’ and ‘T’ were the favorable and unfavorable alleles, respectively. The LOD score of each dot represents a transformed P value, -lg(P). A total of 222 varieties containing ‘C’ and 40 varieties containing ‘T’ were designated C-type and T-type, respectively. From the three years results, we found that the mean disease score of C-type varieties was remarkably decreased compared to the T-type varieties, implying the significant effect of *qSE-6A* on sharp eyespot resistance (**Figure 3A**). For *qSE-7B*, wsnp_Ex_c3501_6407527 was the most significant SNP locus, in which the favorable and unfavorable haplotypes were ‘G’ and ‘A’ halpotypes, respectively. In total, 114 and 148 varieties harbored the resistant ‘G’ and susceptible ‘A’ haplotypes, respectively. The mean disease score of ‘G’ haplotype variety was markedly reduced compared to ‘A’ haplotype variety (**Figure 3B**). In addition, the mean disease score of ‘CG’ haplotype variety (n = 97) harboring favorable alleles in both *qSE-6A* and *qSE-7B* loci was even significantly lower than that of ‘TA’ haplotype variety (n = 22) harboring unfavorable alleles. Based on the difference in mean diseases score, the resistance effects of *qSE-6A, qSE-7B* and *qSE-6A&qSE-7B* were determined to be 59.47, 57.22 and 56.82, respectively, and without the two genes was 63.17, indicating the additive effects of the two QTLs (**Figure 3C**). These findings demonstrate that both *qSE-6A* and *qSE-7B* have significant pyramiding effects on wheat sharp eyespot resistance.

Furthermore, the varieties were mainly located in the middle and lower reaches of the Yangtze River, Huanghuai and southwest wheat growing regions. Only a few were introduced from abroad, which harbored the favorable haplotypes “CG”. The reaction to wheat sharp eyespot in all wheat region was close, except for those from abroad. In addition, all known resistance varieties, such as Shanhongmai, CI12633 and Limai16, belonged to the “CG” haplotypes. Hence, the associated SNPs in the *qSE-6A* and *qSE-7B* regions can be used for marker-assisted breeding program to increase the resistance to wheat sharp eyespot (**Figure 3D**).

## Discussion

The genetic architecture of wheat sharp eyespot resistance remains largely unknown due to the lack of appropriate mapping populations and reliable disease evaluation methods. Here, the wheat sharp eyespot resistance of 262 varieties was evaluated in three years, and the results showed that the overall resistance of the varieties was considerably less, and as low as 3% of these varieties were classified as the resistant. The results of GWAS analysis showed that 15 SNP loci were markedly related to wheat sharp eyespot resistance, and two SNP markers on chromosomes 6A and 7B were repeatedly observed in the three years. Two QTLs were designated and named as *qSE-6A* and *qSE-7B*, and the ‘CG’ haplotype varieties could decrease sharp eyespot disease index by about 20% and 10%, respectively. The QTLs of sharp eyespot resistance on chromosome 6A and 7B were mapped to the wheat reference Chinese spring genome based on their flanking or associated markers. The location region of *qSE-6A* (physical location was 351086145bp) was not overlapped with previous QTLs (physical location was 77690500bp) on chromosome 6A, implying that the *qSE-6A* is a novel sharp eyespot resistance locus.

No studies have been reported on the use of GWAS with high-density SNP markers for identifying loci related to sharp eyespot resistance. To further verify *qSE-6A* and *qSE-7B*, some LD analysis-related populations should be established *via* a backcrossing or hybridization strategy.

One of the advantages of GWAS is that it can be used to analyze the target varieties with favorable/unfavorable alleles, which is unattainable by the LD analysis of two parental alleles. Hence, GWAS data can be successfully adopted in the breeding practices. Based on the results of SNP markers related to sharp eyespot resistance, the suitable parents can be selected, and we also can attain rapid transfer of the target loci by performing genomic breeding and marker-assisted selection. However, it may impractical to enhance the overall resistance of wheat to sharp eyespot through marker-assisted polygenic polymerization, as this disease is controlled by multiple genes. Thus, it is of imperative importance to alter the frequencies of wheat resistant to sharp eyespot during conventional breeding. In this research, we observed that some abroad imported varieties and landraces were significantly more resistant than other region varieties, which may be attributed to the fact that the majority of abroad varieties and landraces harbored “CG” haplotypes in *qSE-6A* and *qSE-7B* regions.

In this study, the two QTLs, *qSE-6A* and *qSE-7B*, were delimited into ≤0.8 Mb regions with only 15 annotated high-confidence SNPs. Thus, the putative genes in some of the QTL regions can be identified based on the wheat reference genome annotation (International Wheat Genome Sequencing Consortium (IWGSC), 2018). The disease resistance mechanism in plants is relatively complex, which involves the activation of disease resistance genes, pathogenesis-related genes, transcription factors, mitogen-activated protein kinase pathways, and recognition of pathogen-associated patterns or effectors. Thus far, 9 resistance genes have been cloned for wheat resistance to sharp eyespot, and the types of resistance genes are diversified, such as *COMT* (Wang et al., 2018), pathogen-induced *ERF1* (Wei et al., 2016), *TaAGC1* (Zhu et al., 2015), *RcCUT1* (Lu et al., 2018), *TaCAD12* (Rong et al., 2016), *PP2A* (Zhu et al., 2018), *TaCPK7-D* (Wei et al., 2016), *TaRCR1* (Zhu et al., 2017), *TaRIM1* (Shan et al., 2016), and *DmAMP1W* (Su et al., 2020). These genes all contributed to sharp eyespot resistance and can serve as potential QTLs for plant disease resistance.

In the QTLs associated with sharp eyespot resistance, 193 genes were annotated, and 5 of them were resistant genes from 2 QTLs (**Supplemental Table 6**). All of the five genes were detected in three years, implying that these genes can be used for identifying causal variants in these QTLs. Based on the transcriptomic data from our previous research, a putative gene that activated by sharp eyespot pathogen was identified in this study. *qSE-7B* on chromosome 7B was significant under the three environments. This QTL region contains a carboxypeptidase gene, *TraesCS7B01G054900.1*, which participates in lignin synthesis. In the future, CRISPR-Cas9-based gene editing will be conducted to validate these candidate genes.

In this study, GWAS analysis was performed to assess sharp eyespot resistance based on 34039 SNPs in 262 wheat accessions. Two QTLs were correlated with sharp eyespot in three years. The two QTLs were stably detected in the three years, and the QTL in chromosome 6A was newly discovered. Functional analysis revealed that 8 core genes were associated with sharp eyespot resistance. The robustness of multilocus GWAS approach in studying wheat sharp eyespot resistance was evaluated. This study allows us to better understand the mechanism of wheat sharp eyespot resistance. The identified QTLs and putative SNPs can be employed to characterize genes related to wheat sharp eyespot resistance and further improve this disease through marker-assisted breeding.

## Data availability statement

The original contributions presented in the study are included in the article/**Supplementary Material**. Further inquiries can be directed to the corresponding author.

## Author contributions

XW and JW: Conceptualization, Supervision, Resources, writing review and editing and Funding acquisition. YZ: Supervision, Resources and Funding acquisition. DW and WJ performed the experiments; ZG and DL provided additional resources and suggestions; RW and DG provided guidance throughout the entire study. All authors contributed to the article and approved the submitted version.

## Supplementary material

Supplementary Table 2 The information of 90K SNP

Supplementary Table 3 Phenotypic variation for sharp eyespt in three years (2018-2020)

Supplementary Table 4 The raw analysis data

Supplementary Table 6 Annotated genes within the identified QTL region
