# Supplementary material for: Identification of new resistance loci against wheat sharp eyespot through genome-wide association study
Source: Front Plant Sci. 2022 Dec 12;13:1056935. doi: 10.3389/fpls.2022.1056935 (PMC9792169; doi:10.3389/fpls.2022.1056935)
Supplement: Supplementary file 1 [file DataSheet_1.docx]

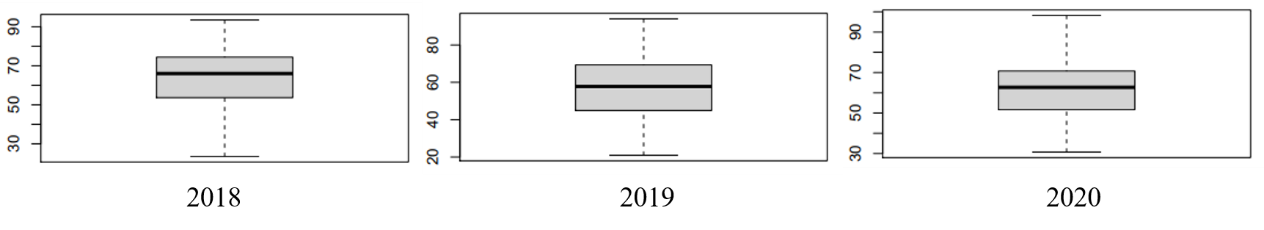


Supplemental Figure 1. Sharp eyespot severity of the natural population in 2018-2020.


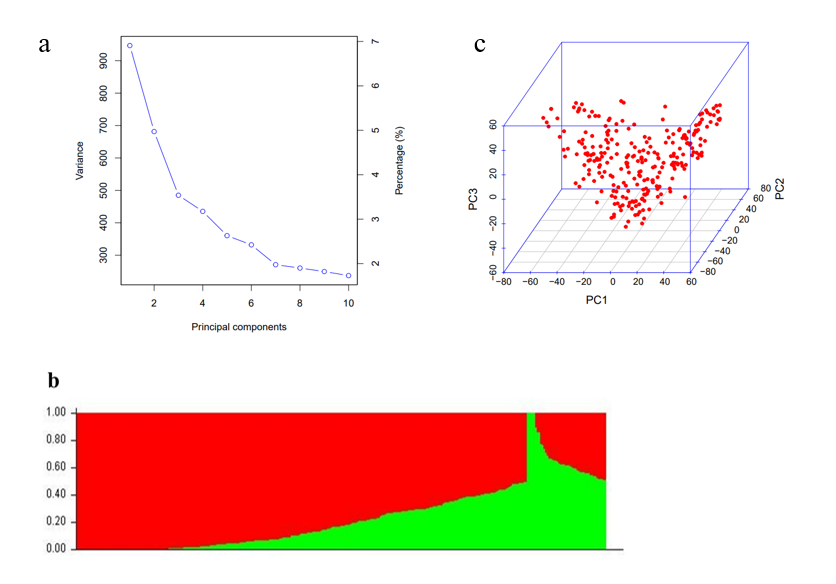


Supplemental Figure 2. (a) *ΔK* Plot containing putative *k* ranging from 2 to 10. (b) Plot of subpopulations *k*=2, represented by two colors i.e. red and green which indicates the proportion of each subpopulation. (c) PCA based on standardized covariance of genotypic data.


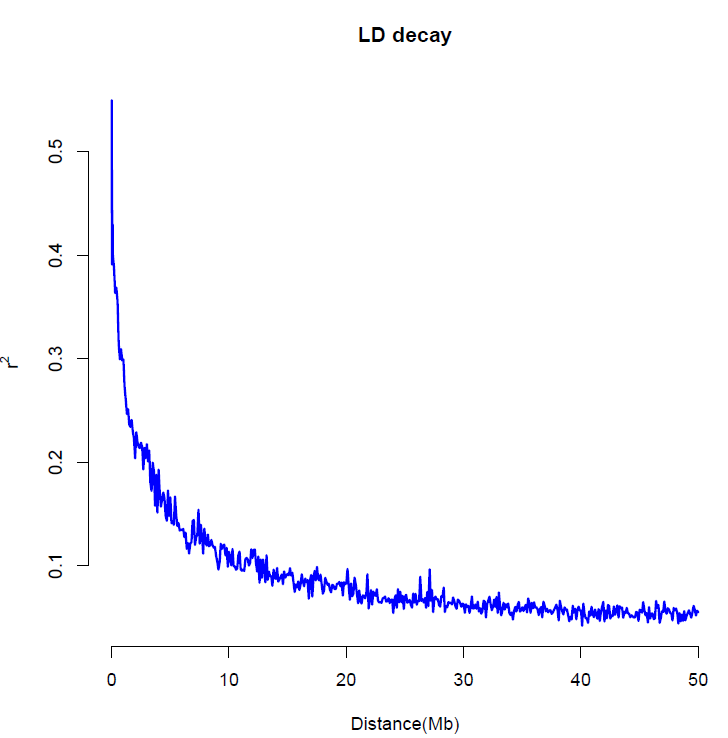


Supplemental Figure 3. The LD decay determined as r^2^ values against the genetic distance between SNP pairs in the whole genome.
